# Prognostic implications of preoperative anemia in urothelial carcinoma: A meta-analysis

**DOI:** 10.1371/journal.pone.0171701

**Published:** 2017-02-09

**Authors:** Fei Luo, Ya-Shen Wang, Yan-Hui Su, Zhi-Hua Zhang, Hong-Hong Sun, Jian Li

**Affiliations:** Department of Urology, Tianjin Union Medical Center, Tianjin, China; University of Nebraska Medical Center, UNITED STATES

## Abstract

The prognostic significance of preoperative anemia (PA) has been identified in various malignancies. However, its predictive role in urothelial carcinoma (UC) remains controversial. The aim of this study was to investigate the prognostic value of PA in UC patients. We performed a meta-analysis of the association between PA and survival outcome in UC patients. Electronic databases were searched up to June 30, 2016. Study characteristics and prognostic data were extracted from each included study. Cancer-specific survival (CSS), recurrence-free survival (RFS), and overall survival (OS) were pooled using hazard ratio (HR) with corresponding 95% confidence intervals (CI). Herein, 12 studies comprising 3815 patients were included in the meta-analysis. There were 1593 (41.76%) patients in the PA group and 2222 (58.24%) in the control group. The overall pooled HRs of PA for CSS, RFS, and OS were significant at 2.21, (95% CI: 1.83–2.65, P_heterogeneity_ = 0.49, I^2^ = 0%), 1.87 (95% CI: 1.59–2.20, P_heterogeneity_ = 0.22, I^2^ = 28%), and 2.04(95% CI: 1.76–2.37, P_heterogeneity_ = 0.36, I^2^ = 9%) respectively. Stratified analyses indicated that PA was a predictor of poor prognosis based on ethnicity, sample size, tumor T stage, G grade, lymphovascular invasion (LVI), concomitant carcinoma in situ (CIS), and follow-up values. Our findings show that PA has negative prognostic effects on the survival outcome (CSS, RFS, and OS) in UC patients and can serve as a useful and cost-effective marker to aid prognosis prediction.

## Introduction

Urothelial carcinoma (UC), which derives from the transitional cells of the urinary tract, is the sixth most common cancer worldwide. It accounts for more than 13,000 deaths annually [1]. Radical nephroureterectomy (RNU) with bladder cuff excision for upper tract urothelial cancer (UTUC), radical cystectomy (RC) with lymph node dissection for muscle-invasive bladder cancer (MIBC), and transurethral resection of bladder tumor (TURBT) for non-muscle invasive bladder cancer (NMIBC) constitute the standard treatment options for UC [2–5]. However, more than 20% of patients experience disease recurrence within 10 years of radical surgery [6–8]. The major pathologic determinants for survival in UC patients, such as tumor stage and surgical margin status, are well established. At present, tissue or blood-based biomarkers, which can also act as prognostic predictors to improve the outcome of UC, have yet to be incorporated into daily clinical practice.

Anemia is the commonest hematological abnormality in cancer patients. An increasing number of studies have shown that preoperative anemia (PA) is a predictor of adverse outcome in surgical patients, especially ones with cancer [9–12]. Up to 60% of cancer patients, including patients with lung, head and neck, and cervical cancer are affected [9, 10, 13–15]. In 2014, Rink et al. found PA to be an independent predictor of cancer recurrence (CR) and cancer-specific mortality (CSM) in UTUC patients treated with RNU [16]. Gierth et al. also demonstrated that PA was significantly associated with poorer oncological outcomes (DR, CSM, and ACM) in MIBC patients undergoing RC [17]. Hence, it seems reasonable to advocate neoadjuvant or adjuvant treatment therapy for UC patients with high risk PA. However, Akdogan et al. reported that PA was not significantly associated with 5-year disease specific and recurrence-free survival in UTUC patients [18].

Therefore, we performed a meta-analysis of 12 studies comprising 3815 UC patients to comprehensively analyze the relationship between PA and outcome for UC patients, as well as to investigate the prognostic value of PA in UC.

## Materials and methods

### Search strategy

**Fig 1:**
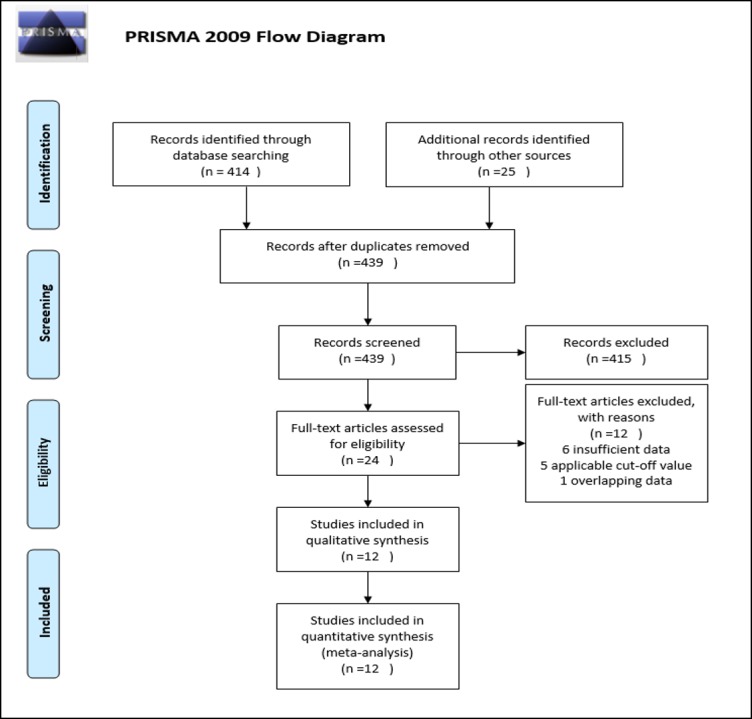
Flow Diagram of the Assessment of Studies Identified for the Meta-analysis. The flow diagram shows eligible publications at each stage of the analysis process. The database search was conducted in July 2016.

A comprehensive search of PubMed, Web of Science and the Cochrane Library database was performed up to June 30, 2016. The MeSH keywords used include: “Preoperative anemia/anaemia”, “PA”,”anemia/anaemia”, “Hemoglobin/Hb”, “bladder cancer/tumor/carcinoma”,”Urothelial cancer/tumor/carcinoma”,”survival”,”mortality”, and”recurrence”. Relevant references cited in the retrieved papers were also screened when they were considered potentially pertinent. If there was more than one publication on the same study population, only the most recent paper was included (Fig 1 and S1 File).

### Selection criteria

The literature was screened in accordance with the following inclusion criteria: 1) English-language publication. (2) UC patients had a pathologically confirmed diagnosis of urological/bladder carcinoma. (3) Anemia was diagnosed in accordance with adjusted criteria [19]. (4) Treatment methods were clearly elucidated. 5) The Kaplan-Meier curve, hazard ratio (HR) or 95% confidence interval (CI) values describing association between overall survival (OS), recurrence-free survival (RFS) and cancer-specific survival (CSS) were available. 6) Relative information on survival and recurrence was provided.

The exclusion criteria were as follows: 1) Reviews, letters, and case reports. 2) Publication did not provide necessary clinical data. 3) Duplicate publications. 4) UC patients did not undergo radical surgical treatment. 5) The hemoglobin levels by which patients were grouped were considered undesirable. 6) Studies on the relationship between PA and non-urological/bladder tumors. Ineligible studies were excluded from the analysis.

### Data extraction and quality assessment

To ensure accuracy and minimize bias, two investigators (FL and YSW) independently determined the eligibility of the studies retrieved from the databases and reference lists. Any discrepancies encountered during data extraction were settled by consensus through discussion. The following details were extracted from each included study: first author, year of publication, country, patient details (number enrolled, age, sex, TNM stage, G grade, carcinoma in situ [CIS] stage, treatment method, and follow-up time), surveillance endpoints (OS, CSS, and RFS), hazard ratio (HR), and the corresponding 95% CI values.

Data from multivariate analyses were superior to that of univariate analyses, and were used for studies in which both were presented; data from univariate analysis were used when multivariate analysis was unavailable. The Tierney methods [20] were used to derive an estimation of the HR and its corresponding 95% CI from the Kaplan-Meier curve if they were absent from the original articles. Two investigators assessed the quality of the eligible studies by using the Newcastle-Ottawa Scale (NOS) [21]. The selection and comparability of the cohorts, and credibility of the outcome data were the main points used to rate the included studies. Disagreement was reevaluated by all the authors until consensus was reached.

### Statistical analysis

The HR and its corresponding 95% CI was used to calculate the pooled effect value for the comparison of OS, CSS, and RFS. The null value of the HR was set at 1. A HR of >1 indicated that patients with PA had a poorer survival outcome; conversely, a HR of <1 (95% CI, not >1) indicated a better survival outcome. The Cochrane Q test and Higgins I^2^ statistic were used to assess the heterogeneity of pooled studies. If the HRs were found to be unacceptably heterogeneous (I^2^ > 50% or P_heterogeneity_ < 0.10), a random effects model was used; otherwise, a fixed effects model was used to calculate the pooled HR. REMAN statistical software (version 5.0) was employed to conduct the meta-analysis. Funnel plots were used to evaluate the risk of publication bias. P < 0.05 was considered statistically significant in this study.

## Results

### Study search and characteristics

As shown in Fig 1, 439 records were identified from the database search and manual retrieval from other sources. After screening the titles and abstracts based on our eligibility criteria, 415 records were deemed ineligible and subsequently removed. 24 full-text articles were retrieved for further evaluation. Of these, 6 did not provide sufficient clinical or endpoint data, 5 used inapplicable cut-off values and 1 was a duplicate. The remaining 12 publications [16–18, 22–30] were included in the meta-analysis.

The 12 included trials were published from 2005 to 2016 and included a total of 3815 patients. 1593 of these belonged to the PA group and 2222 to the control group (normal group). Nine studies comprising 2732 UC patients investigated the CSS between the two groups; 7 studies comprising 2534 patients reported the RFS; and 7 comprising 2754 patients compared the OS of both groups. The curative therapies performed in the studies included RC, RNU, and TURBT, all of which were considered radical. All patients were followed-up in line with a set schedule. The baseline characteristics of the 12 trials are shown in Table 1 and S2 File.

**Table 1:** Main Characteristics of all Studies Included in the Meta-analysis.

First author	Year	No. of patients (all/anemia)	Gender (Male/Female)	Country	Time	location	Treatment	NOS score
HC. Yeh	2016	370(242)	148/222	Taiwan	2000–2013	Upper Tract	Radical Nephroureterectomy	7
JK. Jo	2016	200(119)	176/24	Korea	2003–2014	Bladder	Radical Cystectomy	7
O. Celik	2016	320(118)	268/52	Turkey	2006–2014	Bladder	TURBT	8
T. Schubert	2016	246(64)	182/64	Germany	1999–2009	Bladder	Radical Cystectomy	8
T. Hara	2016	254(109)	207/47	Japan	2001–2010	Bladder	Radical Cystectomy	8
B. Milojevic	2015	238(97)	132/106	Serbia	1999–2013	Upper Tract	Radical Nephroureterectomy	7
M. Gierth	2015	684(269)	551/133	Germany	2001–2011	Bladder	Radical Cystectomy	8
N. Hinata	2015	730(276)	561/169	Japan	2001–2010	Bladder	Radical Cystectomy	8
M. Rink	2014	282(112)	179/103	Germany	1992–2012	Upper Tract	Radical Nephroureterectomy	7
S. Morizane	2013	99(68)	71/28	Japan	1995–2011	Upper Tract	Radical Nephroureterectomy	6
HM. Bruins	2013	320(97)	235/85	Netherlands	1998–2011	Bladder	Radical Cystectomy	7
B. Akdogan	2005	72(22)	59/13	Turkey	1987–2003	Upper Tract	Radical Nephroureterectomy	6

### Survival analysis

**Fig 2:**
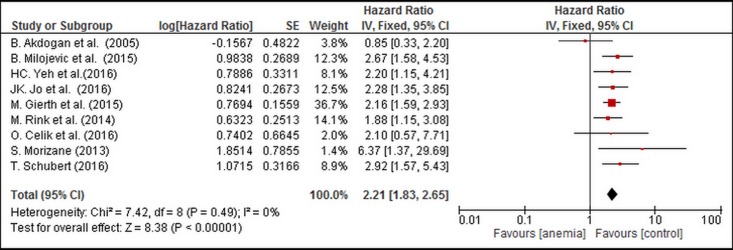
Forest Plot Illustrating the Meta-analysis of the Prognostic Value of PA for CSS.

On pooling the data from 9 of 12 studies, PA was found to be significantly associated with poor CSS in the fixed effects model (combined HR: 2.21, 95% CI: 1.83–2.65). Statistical heterogeneity was not significant. (P_heterogeneity_ = 0.49, I^2^ = 0%) (Fig 2).

The size of the square box is proportional to the weight that each study contributed in the meta-analysis. The diamond represents the overall estimate and CI. The fixed-effects model was used for the meta-analysis.

**Fig 3:**
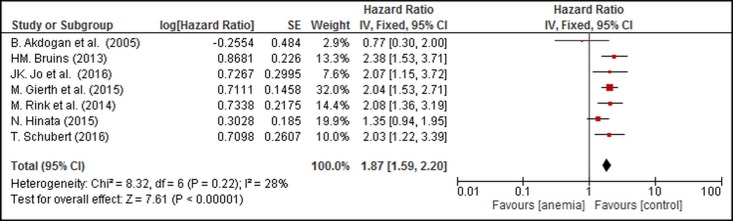
Forest Plot Illustrating the Meta-analysis of the Prognostic Value of PA for RFS in UC Patients.

A meta-analysis of 7 studies (2,534 patients) showed that PA resulted in poorer RFS (combined HR: 1.87, 95% CI: 1.59–2.20, Fig 3). There was no evidence of significant statistical heterogeneity in the analysis (P_heterogeneity_ = 0.22, I^2^ = 28%).

**Fig 4:**
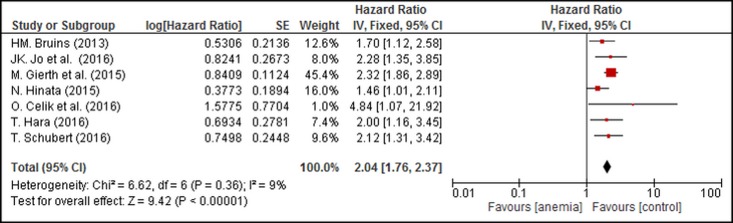
Forest Plot Illustrating the Meta-analysis of the Prognostic Value of PA for Overall Survival (OS) in UC Patients.

Seven of 12 studies assessed the relationship between PA and OS. The pooled data showed that patients with PA exhibited significantly lower OS than patients without PA (combined HR: 2.04, 95% CI: 1.76–2.37, P_heterogeneity_ = 0.36, I^2^ = 9%) (Fig 4).

### Subgroup analysis

**Fig 5:**
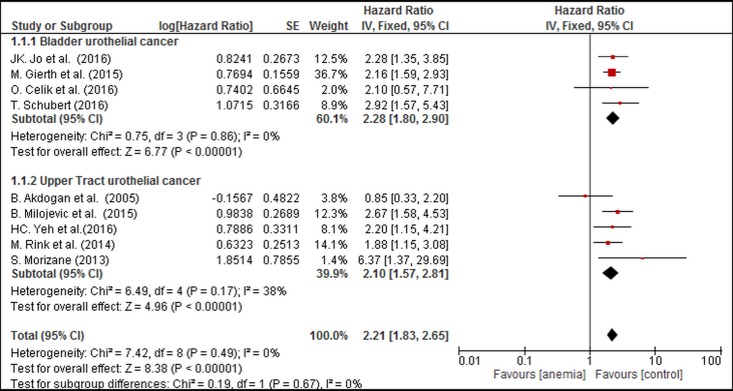
Forest Plot Illustrating the Subgroup Analysis of the Prognostic Value of PA for CSS in BC and UTUC.

A subgroup analysis based on UC location was performed to investigate the association between PA and worse prognosis. We found that the PA group had a lower CSS than the control group in both subgroups (Fig 5; Bladder cancer: HR = 2.28, 95% CI: 1.80–2.90, P_heterogeneity_ = 0.86, I^2^ = 0% vs. UTUC: HR = 2.10, 95% CI: 1.57–2.11, P_heterogeneity_ = 0.17, I^2^ = 38%). In addition, subgroup analyses revealed that PA was linked to worse CSS and OS in all subgroups based on ethnicity (Asian and Caucasian), sample size (number of patients, >300 and ≤300), tumor T stage (T3-4, ≤40% and >40%), G grade (G3, >50% and ≤50%), lymphovascular invasion LVI (≤20% and >20%), concomitant CIS (≤20% and >20%), and follow-up values (period and end time, respectively). The subgroup analyses for RFS had similar results, except that in the groups with upper tract tumors and G3 of ≤50%. All the above-mentioned results are detailed in Table 2(S3 File) and supplement Figures A-Y in S5 File.

**Table 2:** Results of the subgroup analysis of the prognostic significance of PA.

	CSS							RFS							OS						
Variables	No. Study	HR	95%CI	PZ	I2,%	Pheterogeneity	Model	No. Study	HR	95%CI	PZ	I2,%	Pheterogeneity	Model	No. Study	HR	95%CI	PZ	I2,%	Pheterogeneity	Model
Location																					
Upper Tract	5	2.1	1.57–2.81	<0.00001	38	0.17	Fixed	2	1.4	0.54–3.62	0.49	71	0.06	Random	0	-	-	-	-	-	-
Bladder	4	2.28	1.80–2.90	<0.00001	0	0.86	Fixed	5	1.9	1.56–2.31	<0.00001	16	0.32	Random	7	2.04	1.76–2.37	<0.00001	9	0.36	Fixed
Ethnic																					
Asian	3	2.41	1.62–3.57	<0.0001	0	0.44	Fixed	2	1.52	1.12–2.07	0.008	31	0.23	Fixed	3	1.76	1.35–2.30	<0.0001	6	0.34	Fixed
Caucasian	6	2.15	1.75–2.65	<0.00001	10	0.35	Fixed	5	2.02	1.68–2.45	<0.00001	11	0.35	Fixed	4	2.19	1.83–2.61	<0.00001	0	0.43	Fixed
No. of patients																					
>300	3	2.16	1.65–2.83	<0.00001	0	1	Fixed	2	1.69	1.13–2.51	0.01	67	0.08	Random	3	2.02	1.31–3.12	0.002	65	0.06	Random
≤300	6	2.25	1.74–2.89	<0.00001	32	0.19	Fixed	5	2.01	1.57–2.58	<0.00001	11	0.34	Random	4	1.98	1.55–2.52	<0.00001	0	0.83	Random
Stage																					
T3-4≤40%	4	1.94	1.36–2.79	0.0003	12	0.33	Fixed	3	1.79	1.04–3.06	0.03	55	0.11	Random	3	1.99	1.44–2.74	<0.0001	6	0.34	Fixed
T3-4>40%	5	2.31	1.86–2.87	<0.00001	0	0.5	Fixed	4	1.83	1.49–2.26	<0.00001	19	0.29	Random	4	2.06	1.74–2.43	<0.00001	33	0.22	Fixed
Grade																					
G3>50%	3	2.22	1.60–3.10	<0.00001	0	0.55	Random	3	1.71	1.34–2.18	<0.0001	30	0.24	Fixed	3	1.74	1.35–2.26	<0.0001	0	0.41	Fixed
G3≤50%	4	2.15	1.05–4.38	0.04	52	0.1	Random	1	0.77	0.30–2.00	0.6	-	-	Fixed	1	4.48	1.07–21.92	0.04	-	-	Fixed
LVI																					
LVI≤20%	4	2.5	1.82–3.43	<0.00001	0	0.63	Fixed	2	2.26	1.59–3.22	<0.00001	0	0.71	Fixed	2	1.91	1.37–2.64	0.0001	0	0.39	Fixed
LVI>20%	3	2.19	1.72–2.78	<0.00001	0	0.55	Fixed	4	1.84	1.53–2.21	<0.00001	19	0.29	Fixed	4	2.06	1.74–2.43	<0.00001	33	0.22	Fixed
CIS(+)																					
≤20%	4	2.18	1.55–3.05	<0.00001	0	0.52	Fixed	2	2.08	1.47–2.93	<0.0001	0	0.98	Fixed	2	2.47	1.51–4.06	0.0003	0	0.36	Random
>20%	2	2.29	1.74–3.01	<0.00001	0	0.39	Fixed	2	2.04	1.59–2.61	<0.00001	0	1	Fixed	3	1.96	1.46–2.64	<0.00001	55	0.11	Random
Follow-up Time																					
<50months	5	2.42	1.86–3.15	<0.00001	0	0.55	Fixed	3	2.06	1.55–2.75	<0.00001	0	1	Random	3	2.13	1.58–2.87	<0.00001	0	0.94	Random
≥50months	3	1.98	1.49–2.63	<0.00001	40	0.19	Fixed	4	1.7	1.20–2.41	0.003	61	0.05	Random	4	1.92	1.42–2.60	<0.0001	53	0.09	Random
End of Follow-up																					
after 2011	6	2.15	1.74–2.67	<0.00001	0	0.5	Fixed	3	1.91	1.49–2.45	<0.00001	47	0.15	Fixed	3	2.34	1.92–2.87	<0.00001	0	0.64	Fixed
before 2011	3	2.37	1.63–3.45	<0.00001	30	0.24	Fixed	4	1.84	1.49–2.28	<0.00001	34	0.21	Fixed	4	1.73	1.39–2.16	<0.00001	0	0.62	Fixed

### Publication bias

**Fig 6:**
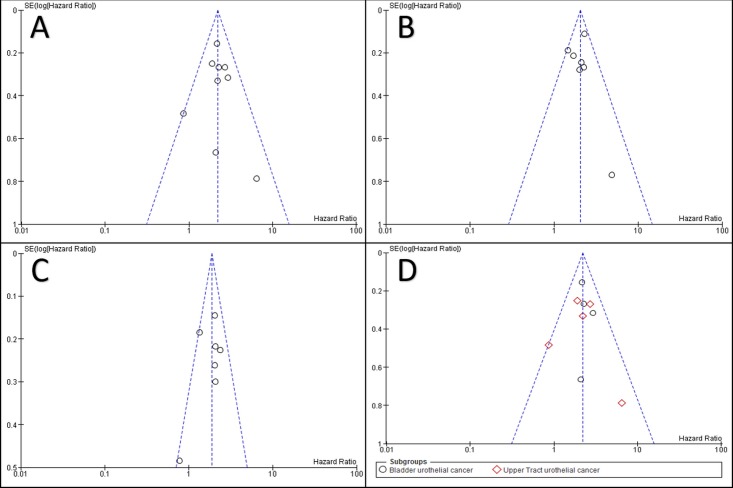
Funnel Plot Illustrating the Lack of Publication Bias in the Meta-analysis for CSS (A), RFS (B), OS (C), and the CSS Subgroup Analysis Comparing BC and UTUC (D).

Fig 6A–6D show the funnel plots used to assess publication bias in the included studies. There was no obvious publication bias for CSS, RFS, or OS in our meta-analysis.

## Discussion

This meta-analysis of 12 studies comprising 3815 participants was performed to estimate the prognostic effect of PA on UC after radical curative therapy. Our results demonstrated that PA is significantly associated with worse CSS, RFS, and OS. We have also found that PA has a substantial prognostic value for CSS in both UTUC and BC, and this was confirmed by the results of the subgroup analysis. In addition, we also performed stratified analyses of CCS, RFS, and OS according to ethnicity, sample size, tumor T stage, G grade, LVI, concomitant CIS, and follow-up values. All these findings suggest that PA was an effective prognostic predictor in UC patients treated with radical surgery. Anemia has a prevalence of up to 50% in UC patients [31, 32]. Anemia, as a potentially cytokine-mediated disorder, may develop long before the diagnosis of UC is made [33]. The mechanisms that could potentially explain the correlation between PA and poor survival outcome in patients with UC remain complex and unclear. It is well known that anemia can result in hypoxia in the tumor microenvironment, which may lead to instability and modification of hypoxia-induced genes such as VEGF, p53, and HIF-1 [34, 35]. There is a growing body of evidence from around the world that chronic inflammation contributes to cancer progression and cancer-related deaths [36, 37]. Furthermore, research has shown that the increased level of VEGF in tumor cells of PA patients promotes angiogenesis, and tumor cells with p53 mutations may lose their apoptotic potential in hypoxic microenvironments [38–40]. In addition, HIF-1 upregulated by anemia can induce tumor cell migration and facilitate metastasis [41, 42]. Increasingly, more “new” cell variants with various phenotypes are emerging, and anemia can also be considered to promote “microaggressive and metastatic” disease [43]. As such, the results of our meta-analysis suggest that UC patients with PA may benefit from neoadjuvant or adjuvant treatment within the perioperative period.

Overexpression of certain inflammatory cytokines can also reduce the survival time of red blood cells, suppress erythroid progenitor cells, and arrest the bone marrow response to erythropoietin. This may aggravate the anemia experienced by UC patients [24, 44, 45]. This correlation may also be responsible for enhanced tumor growth, progression, and metastasis. Therefore, tumor patients who are anemic at diagnosis may be at higher risk of metastasis and have a poorer survival outcome.

Histopathological data such as tumor stage, grade, and LVI status were useful for prognosis prediction and treatment decision making for UC patients; however, they were unavailable before the operation [46]. In the present study, we found that preoperative hemoglobin level was a promising biomarker. Owing to the easy measurement in daily practice, hemoglobin level combined with other laboratory predictors reported such as thrombocyte and C-reactive protein levels could predict prognosis as an important supplementary element in UC patients treated with radical surgery[47]. As already known, perioperative blood transfusion (PBT) or erythropoiesis-stimulating agents (ESAs) could alleviate PA and patient discomfort. However, several studies showed that perioperative blood transfusion was associated with poor prognosis [48]; therefore, validation with a large cohort is required to yield a better understating of the alleviation of PA. It may be argued that our meta-analysis utilized several cut-off values to define anemia, which may result in inaccurate outcomes. However, we have excluded several studies that utilized undesirable standards. In addition, we have confirmed that the PA threshold used in all included studies have been adjusted and are applicable to each specific study population[19]. Finally, Gierth et al. has illustrated that the different classifications for BC patients treated with RC do not differ significantly in their prognostic impact [17]. Therefore, our results are assuredly reasonable and clinical meaningful.

To the best of our knowledge, this study is the first meta-analysis relating to the prognostic impact of PA in UC patients. However, our results should be viewed cautiously owing to several limitations. Firstly, analyses were limited by the amount of data available in each included study. Some HRs were indirectly extracted from survival curves or other relevant data, which may prove less reliable than that taken directly from the original studies. Secondly, although strict inclusion criteria were set, patients from different studies may differ in their baseline characteristics (ethnicity, region, disease stage and treatment regimens), which are also potentially confounding factors. In addition, we did not assess the potential effects of other factors. Owing to these limitations, the value of PA as a prognostic indicator in UC still requires confirmation by well-designed trials in the future.

## Conclusions

Our study has shown that UC patients with PA have significantly poorer CSS, RFS, and OS after undergoing radical curative therapy. PA, in combination with other important biomarkers, may useful as an effective predictor of survival prognosis of UC patients.

## Supporting information

S1 File PRISMA 2009 flow diagram. (DOC)

S2 File PRISMA 2009 checklist. (DOC)

S3 File Main Characteristics of all Studies Included in the Meta-analysis. (XLSX)

S4 File Results of the subgroup analysis of the prognostic significance of PA. (XLSX)

S5 File Forest Plot of the subgroup analysis of the prognostic significance of PA. (DOCX)
