# Supplementary material for: Prognostic implications of preoperative anemia in urothelial carcinoma: A meta-analysis
Source: PLoS One. 2017 Feb 9;12(2):e0171701. doi: 10.1371/journal.pone.0171701 (PMC5300162; doi:10.1371/journal.pone.0171701)
Supplement: S1 File — (DOC) [file pone.0171701.s001.DOC]

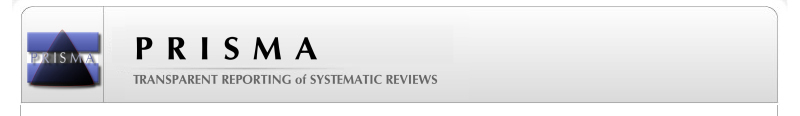
**PRISMA 2009 Flow Diagram**

**Screening**

**Included**

**Eligibility**

**Identification**

Records identified through database searching
(n = 414 )

Additional records identified through other sources
(n =25 )

Records after duplicates removed
(n =439 )

Records screened
(n =439 )

Records excluded
(n =415 )

Full-text articles assessed for eligibility
(n =24 )

Full-text articles excluded, with reasons
(n =12 )

6 insufficient data

5 applicable cut-off value

1 overlapping data

Studies included in qualitative synthesis
(n =12 )

Studies included in quantitative synthesis (meta-analysis)
(n =12 )
