# Supplementary material for: Prognostic implications of preoperative anemia in urothelial carcinoma: A meta-analysis
Source: PLoS One. 2017 Feb 9;12(2):e0171701. doi: 10.1371/journal.pone.0171701 (PMC5300162; doi:10.1371/journal.pone.0171701)
Supplement: S5 File — (DOCX) [file pone.0171701.s005.docx]

**Forest Plot Illustrating the Subgroup Analysis of the Prognostic Value of PA for**

**CSS, RFS, and OS**


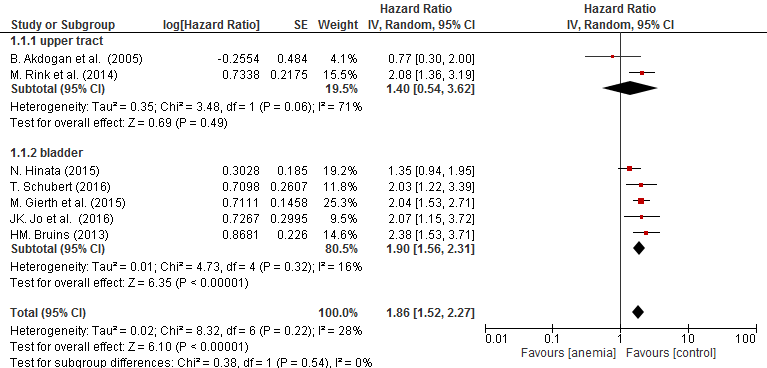


Figure A. Forest Plot of the Prognostic Value of PA for RFS in BC and UTUC patients


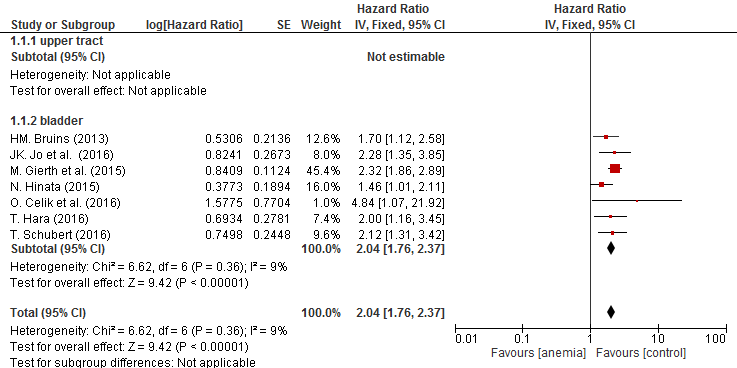


Figure B. Forest Plot of the Prognostic Value of PA for OS in BC and UTUC patients


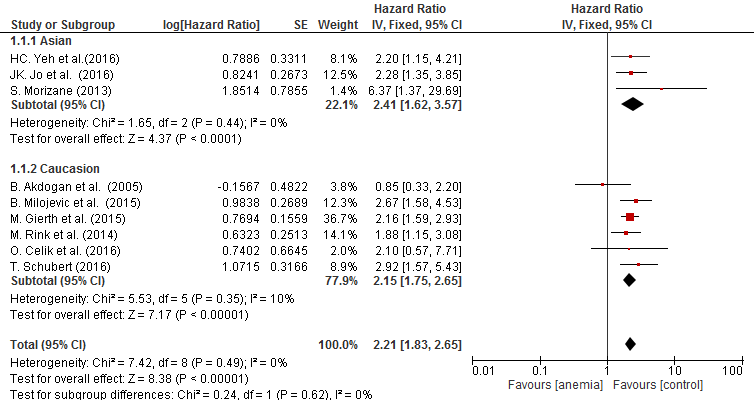


Figure C. Forest Plot of the Prognostic Value of PA for CCS in Asian and Caucasian population


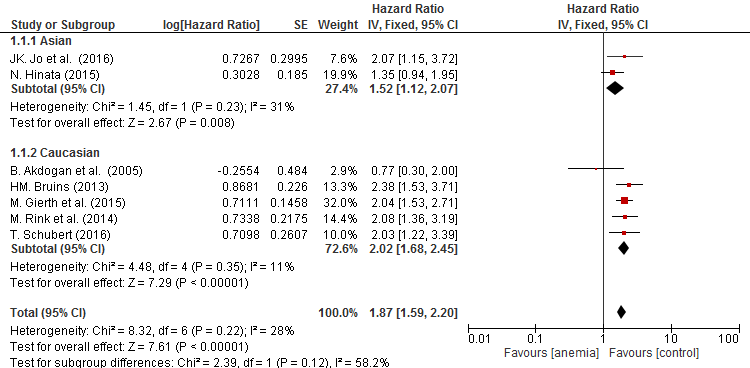


Figure D. Forest Plot of the Prognostic Value of PA for RFS in the Asian and Caucasian population


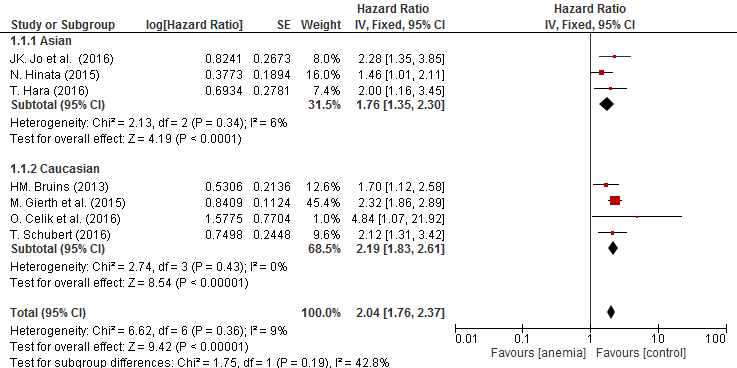


Figure E. Forest Plot of the Prognostic Value of PA for OS in the Asian and Caucasian population


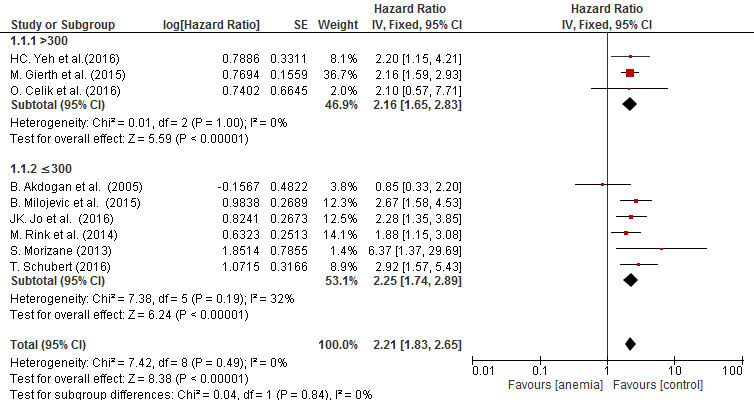


Figure F. Forest Plot of the Prognostic Value of PA for CCS within different sample sizes.


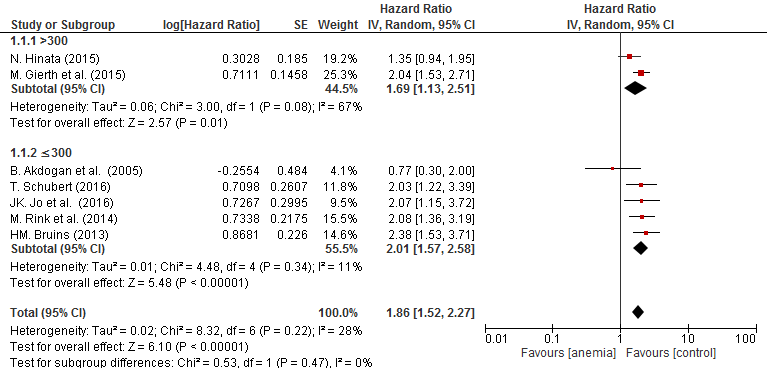


Figure G. Forest Plot of the Prognostic Value of PA for RFS within different size of sample.


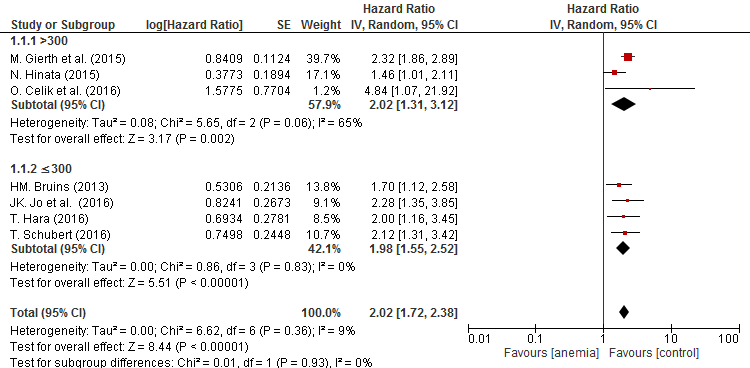


Figure H. Forest Plot of the Prognostic Value of PA for OS within different sample sizes.


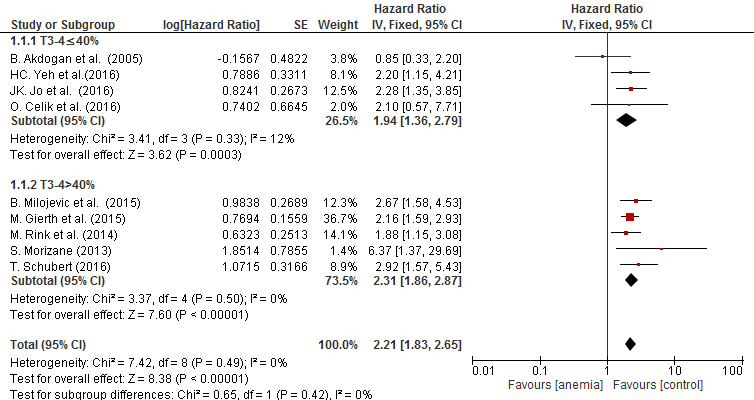


Figure I. Forest Plot of the Prognostic Value of PA for CCS with high and low T stage.


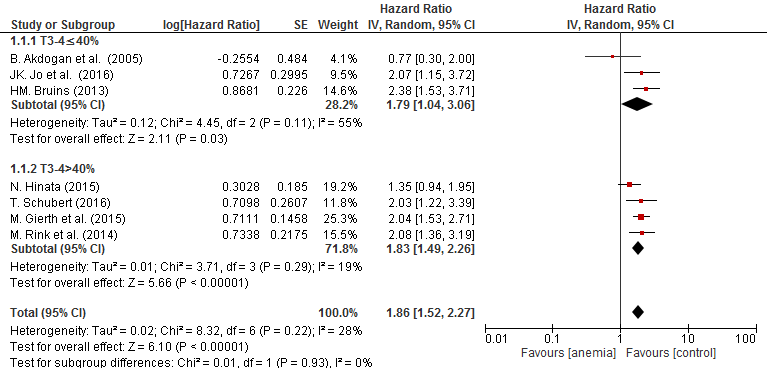


Figure J. Forest Plot of the Prognostic Value of PA for RFS with high and low T stage.


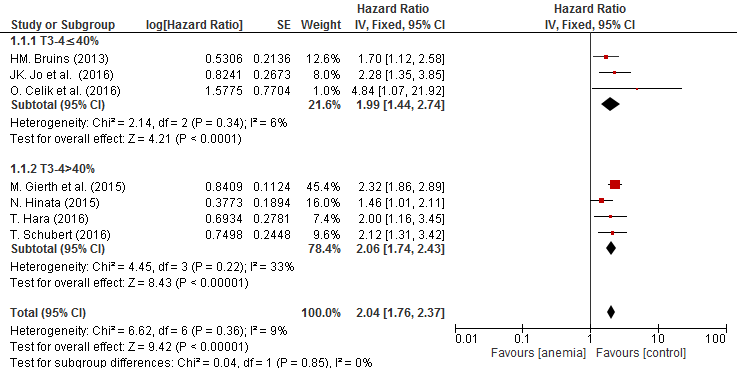


Figure K. Forest Plot of the Prognostic Value of PA for OS with high and low T stage.


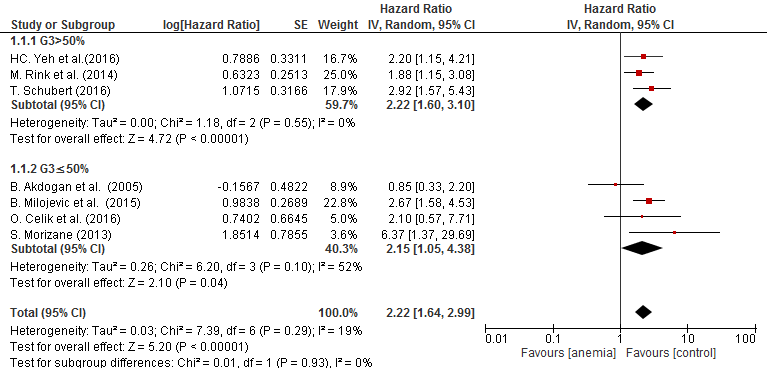


Figure L. Forest Plot of the Prognostic Value of PA for CCS with high and low G grade.


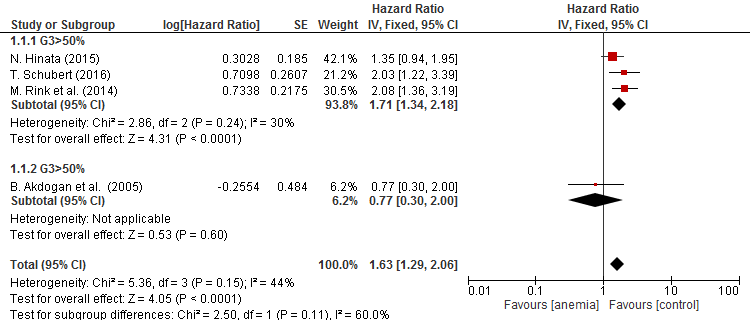


Figure M. Forest Plot of the Prognostic Value of PA for RFS with high and low G grade.


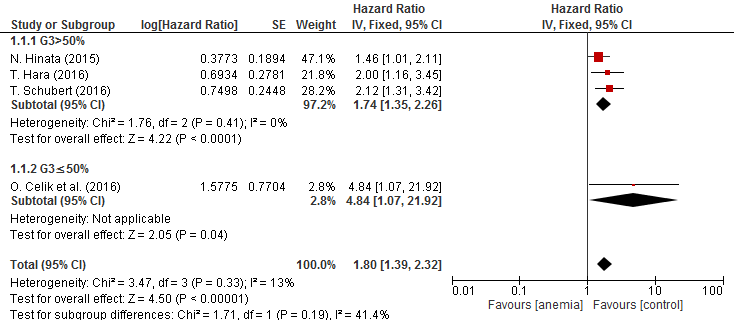


Figure N. Forest Plot of the Prognostic Value of PA for OS with high and low G grade.


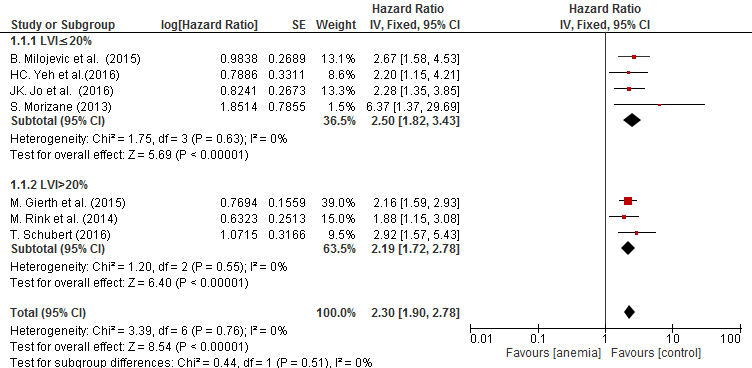


Figure O. Forest Plot of the Prognostic Value of PA for CCS in patients with distinct lymphovascular invasion.


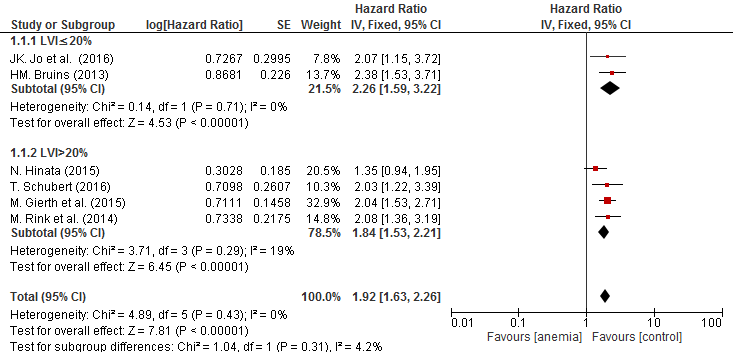


Figure P. Forest Plot of the Prognostic Value of PA for RFS in patients with distinct lymphovascular invasion.


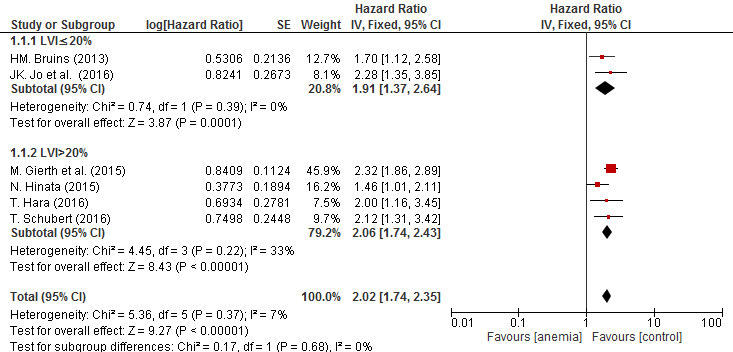


Figure Q. Forest Plot of the Prognostic Value of PA for OS in patients with distinct lymphovascular invasion.


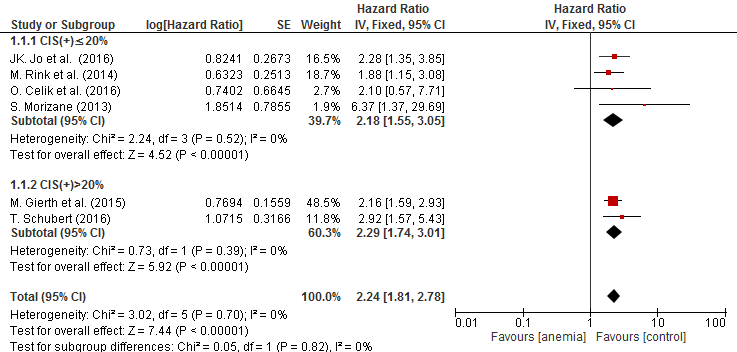


Figure R. Forest Plot of the Prognostic Value of PA for CCS in patients presenting with CIS (+).


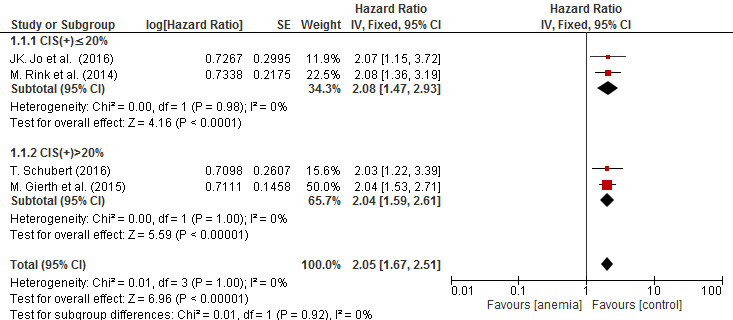


Figure S. Forest Plot of the Prognostic Value of PA for RFS in patients presenting with CIS (+).


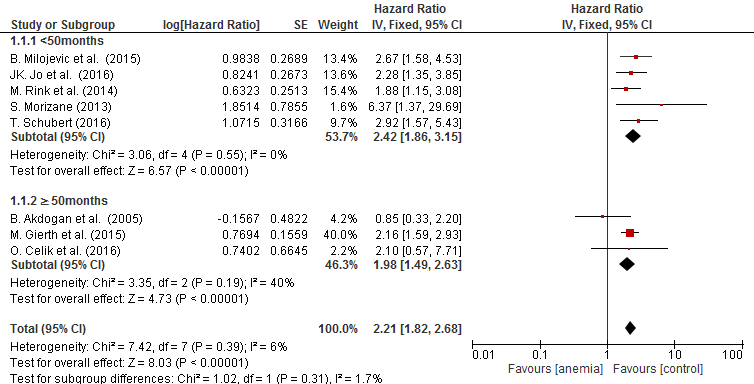


Figure T. Forest Plot of the Prognostic Value of PA for CCS with different follow-up periods.


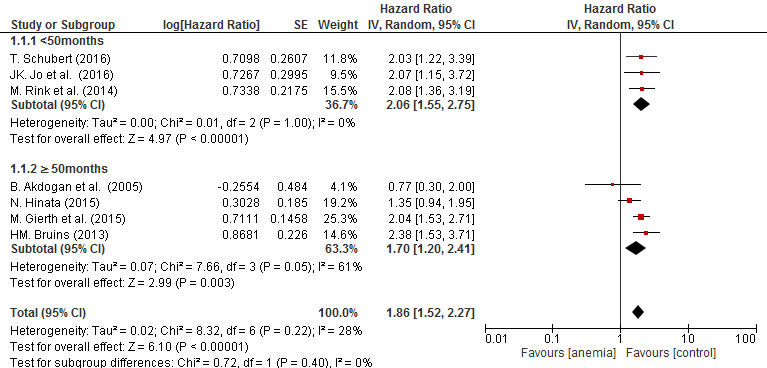


Figure U. Forest Plot of the Prognostic Value of PA for RFS with different follow-up periods.


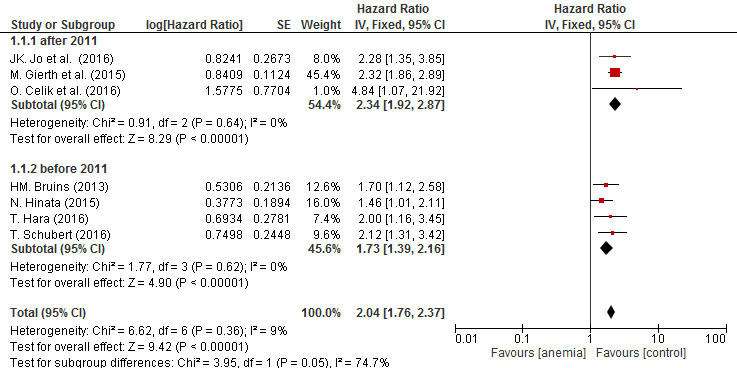


Figure V. Forest Plot of the Prognostic Value of PA for OS with different follow-up periods.


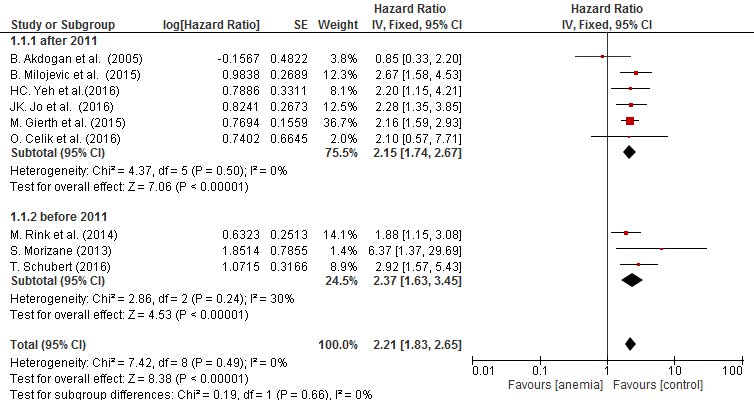


Figure W. Forest Plot of the Prognostic Value of PA for CCS with end time of follow-up before or after 2011.


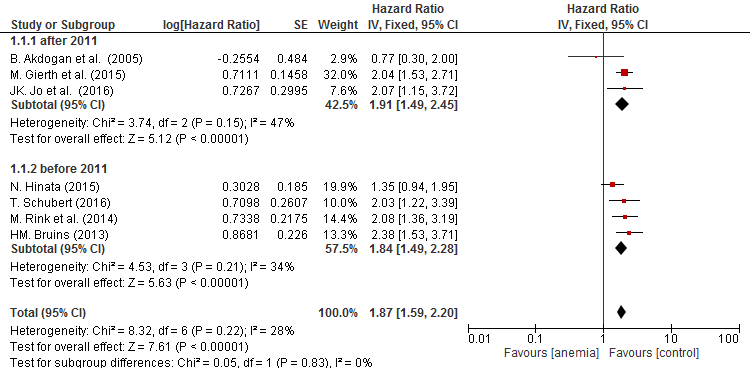


Figure X. Forest Plot of the Prognostic Value of PA for RFS with a follow-up end time before or after 2011.


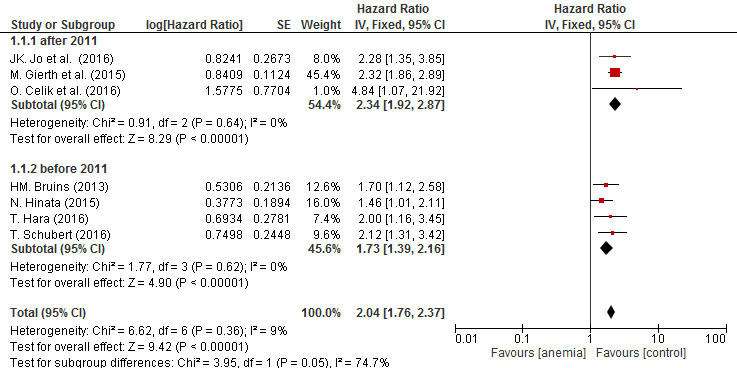


Figure Y. Forest Plot of the Prognostic Value of PA for OS with a follow-up end time before or after 2011.
